# Quality of life and pain in premenopausal women with major depressive disorder: The POWER Study

**DOI:** 10.1186/1477-7525-4-2

**Published:** 2006-01-18

**Authors:** Jill M Hartman, Ann Berger, Karen Baker, Jacques Bolle, Daniel Handel, Andrew Mannes, Donna Pereira, Diane St Germain, Donna Ronsaville, Nina Sonbolian, Sara Torvik, Karim A Calis, Terry M Phillips, Giovanni Cizza, the P.O.W.E.R. (Premenopausal, Osteoporosis, Women, Alendronate, Depression) Study Group

**Affiliations:** 1 NIH Clinical Center, National Institutes of Health, Bethesda, MD, USA; 2 National Institute of Mental Health, National Institutes of Health, Bethesda, MD, USA; 3 National Institutes of Digestive and Kidney Disorders, National Institutes of Health, Bethesda, MD, USA; 4 Ultramicro Analytical Immunochemistry Resource, Division of Bioengineering & Physical Science, Office of Research Services, National Institutes of Health, Bethesda, MD, USA

## Abstract

**Background:**

Whereas it is established that organic pain may induce depression, it is unclear whether pain is more common in healthy subjects with depression. We assessed the prevalence of pain in premenopausal women with major depression (MDD). Subjects were 21- to 45-year-old premenopausal women with MDD (N = 70; age: 35.4 +/- 6.6; mean +/- SD) and healthy matched controls (N = 36; age 35.4 +/- 6.4) participating in a study of bone turnover, the P.O.W.E.R. (Premenopausal, Osteopenia/Osteoporosis, Women, Alendronate, Depression) Study.

**Methods:**

Patients received a clinical assessment by a pain specialist, which included the administration of two standardized forms for pain, the Brief Pain Inventory – Short Form, and the Initial Pain Assessment Tool, and two scales of everyday stressors, the Hassles and Uplifts Scales. In addition, a quality-of-life instrument, the SF-36, was used. The diagnosis of MDD was established by a semi-structured interview, according to the DSM-IV criteria. Substance P (SP) and calcitonin-gene-related-peptide (CGRP), neuropeptides which are known mediators of pain, were measured every hour for 24 h in a subgroup of patients (N = 17) and controls (N = 14).

**Results:**

Approximately one-half of the women with depression reported pain of mild intensity. Pain intensity was significantly correlated with the severity of depression (r^2 ^= 0.076; P = 0.04) and tended to be correlated with the severity of anxiety, (r^2 ^= 0.065; P = 0.07), and the number of depressive episodes (r^2 ^= 0.072; P = 0.09). Women with MDD complained of fatigue, insomnia, and memory problems and experienced everyday negative stressors more frequently than controls. Quality of life was decreased in women with depression, as indicated by lower scores in the emotional and social well-being domains of the SF-36. SP (P < 0.0003) and CGRP (P < 0.0001) were higher in depressed subjects.

**Conclusion:**

Women with depression experienced pain more frequently than controls, had a lower quality of life, and complained more of daily stressors. Assessment of pain may be important in the clinical evaluation of women with MDD. SP and CGRP may be useful biological markers in women with MDD.

## Background

Major Depressive Disorder (MDD), a prevalent and disabling condition [1], is characterized by psychological and physical manifestations. Patients with MDD may present with somatic symptoms, including aches and pain. Pain due to an underlying medical condition may induce or exacerbate depressive symptoms [2-8]. However, the prevalence of pain in patients presenting with MDD, in whom pain was not the primary complaint, has not been well characterized.

There are few studies of pain prevalence in patients presenting with MDD in whom pain was not the primary complaint [9], whereas it is well established that pain and depression are sometimes so intertwined that it is difficult to determine whether bodily pain precipitated depression or whether the depressive symptoms resulted in pain [10,11]. Furthermore, pain and depression are both associated with insomnia and altered sleep quality, and impaired quality of life [12].

The aims of the current study were: 1) to establish the prevalence of pain and associated symptoms in premenopausal women with MDD compared to healthy controls; 2) to determine whether there is a relationship between pain intensity and the clinical features of depression; and 3) to examine the relationship of neuropeptides (Substance P (SP) and Calcitonin-Gene-Related-Peptide (CGRP)) to major depression.

## Methods

### Study design

This study was done as part of the P.O.W.E.R. project (Premenopause, Osteopenia/Osteoporosis, Women, Alendronate, Depression), a prospective study of bone turnover in premenopausal women, ages 21 to 45 years with MDD. The P.O.W.E.R. Study consisted of: 1) a longitudinal comparison of BMD in women with MDD and controls (Natural History Arm) and: 2) a randomized, double-blind, placebo-controlled 12 month trial of alendronate in women with MDD and moderate osteopenia (Clinical Trial Arm). Women with MDD and low bone mineral density were randomized to 70 mg of alendronate or matching placebo tablets orally once a week (MRL, NJ). In addition, women in the clinical trial arm received 500 mg daily of elemental calcium and 400 IU of vitamin D. [13]. The Scientific Review Board and the Institutional Review Board of the National Institute of Mental Health approved this study. A written informed consent was obtained from all participants.

### Study subjects

Women were enrolled if they met DSM-IV criteria for MDD and if they had experienced a depressive episode in the preceding three years. The Standard Clinical Interview for DSM-IV Diagnosis (SCID) was used to diagnose major depression and also to screen the control subjects for depression. Severe depression with suicidal risk, schizophrenia, and schizoaffective, eating or bipolar disorders were among the exclusionary criteria. The current clinical severity of depression and anxiety were measured by the Hamilton scale for depression, 24-item version, (HAMD) and the Hamilton scale for anxiety (HAMA), respectively. Pharmacological and non-pharmacological treatments for depression were allowed. Patients with anxiety disorders or a past history of alcohol or drug dependence in remission for five years were eligible. Healthy controls were matched with subjects with MDD based on age ± 3.0 and body mass index (BMI) ± 2.0. Except for two pairs, the rest were matched by race as well.

We report data on 70 women with MDD and 36 controls, a portion (106/133) of the participants of the P.O.W.E.R. study who received an evaluation by the Pain and Palliative Care Service (PPCS) as part of the study protocol. This evaluation was based purely upon practical considerations (evaluator availability and time constraints) rather than patient preference. All subjects who were offered an evaluation accepted it. All medications were recorded, including over-the-counter medications and alternative treatments. Medical history, physical examination, and screening evaluation (electrocardiogram, serum pregnancy test; tests of hematological, thyroid, liver, and renal function) indicated that patients and controls were in good general health.

### Evaluation for pain and related symptoms

Participants received a standardized clinical assessment by trained members of the PPCS team. This assessment included an inquiry about the use of medications and herbal therapies, a list of 30 symptoms usually associated with pain, medical history, and lifestyle information.

The clinical characteristics of the subjects' current pain were assessed. The Brief Pain Inventory – Short Form (BPI-SF) [14] and the Initial Pain Assessment Tool (IPA) were used [15]. The BPI-SF inquires as to the location and intensity of pain during daily life. The BPI-SF utilizes a rating scale of 0 (no pain) to 10 (worst pain possible) and is administered by the evaluator. The BPI asks whether pain over the past 24 hours has interfered with various activities. The IPA, which is a self-administered form, assesses location of pain but relies on the participant's choice of words in describing the way pain affects daily life.

Subjects with MDD may differently perceive minor stresses and pleasures that characterize everyday life. To characterize the role of such factors, we used the Hassles and Uplifts Scales, two validated scales of everyday stressors that focus on relatively minor events of everyday life [16,17]. The Hassles Scale consists of 117 items covering various areas such as work, health, family, friends, the environment, practical considerations, and chance occurrences. The Uplifts Scale includes 135 items reflecting positive daily experiences.

In addition, we administered the SF-36 (Short Form-36) to assess health-related quality of life. This is a validated, self-administered, 36-item questionnaire that generates scores across eight dimensions of health, assessing physical well being (physical functioning, role-physical, bodily pain, general health), as well as emotional and social well being (vitality, social functioning, role-emotional, and mental health) [18]. The SF-36 includes two summary measures: physical component summary (PCS) and mental component summary (MCS) [19].

### Analytical measurements

After one night of acclimation, starting at 08:00, blood samples were taken at 1-hour intervals for 24 hours. SP and CGRP were measured by one of us (T.M.P.) in plasma without knowledge of group allocation using recycling immunoaffinity chromatography, as previously described [20], with recovered analyte quality control by time-of-flight mass spectrometry. Plasma ACTH, cortisol, and urinary free cortisol (UFC) were measured as previously reported [13].

### Data analysis

Differences between subjects with depression and controls were analyzed for continuous variables by t test, and for categorical variables by Chi-Square and Fisher's Exact tests. The relationship between pain intensity, as reflected by the Brief Pain Inventory, and clinical severity of depression (as measured by the HAMD) and anxiety (as measured by the HAMA) was analyzed by single regressions. Instat software was used for all analyses (GraphPad Software Inc., San Diego, CA).

Chronolab software (available from Universidad de Vigo, Spain, Bioengineering & Chronobiology Laboratory, ) was used for cosinor analyses. All results are expressed as mean +/- standard deviation. A P-value of less than 0.05 was considered significant.

## Results

### Demographic and clinical characteristics of study subjects

Subjects with depression and controls had similar age and race; BMI was however slightly higher in women with MDD (Table 1). On average, subjects with depression were mildly depressed and mildly anxious at the time of evaluation, as indicated by HAMD and HAMA scores; however, they had a long standing history of depression of approximately 6 cumulative years and an average of four episodes of depression. Of the 69 patients with MDD for whom depression scores were available, 8 had a Hamilton Depression score of more than 18, indicating moderate to severe depression, at the time of their evaluation. The remainder had a Hamilton score of 18 or less. All subjects had had an episode of major depression within the past three years. Consistent with their mild depression at the time of evaluation, UFC was within normal limits and not different from controls. Similarly, ACTH and cortisol, measured every hour for 24 hours, exhibited a normal rhythmicity and were not different from controls (data not shown). Depressed subjects used more than twice as many medications (excluding oral contraceptives) on a daily basis as did controls (Table 2).

**Table 1:** Baseline clinical characteristics of participants

	**Controls n = 36**	**MDD n = 70**	**P**
Age (years)	35.4 +/- 6.4	35.4 +/- 6.6	n.s.
Race			
Caucasian	91.7%	91.4%	n.s.
Other	8.3%	8.6%	n.s.
BMI (kg/m^2^)	24.4 +/- 3.7	26.6 +/- 6.4 (n = 69)	n.s.
Urinary-free cortisol (nmol/L)	170.9 +/- 83.4 (n = 30)	141.4 +/- 55.4 (n = 28)	n.s.
Hamilton Scale for Anxiety	1.2 +/- 1.7 (n = 35)	7.0 +/- 5.3 (n = 68)	**<0.0001**
Hamilton Scale for Depression	1.4 +/- 1.9 (n = 34)	9.4 +/- 7.7 (n = 69)	**<0.0001**
Depressive Episode Questionnaire	n/a	(n = 53)	
Age at Onset of Depression	n/a	18.3 +/- 8.6	n.s.
# of Depressive Episodes	n/a	4.4 +/- 2.7	n.s.
Cumulative Duration of Depression (months)	n/a	71.6 +/- 84.2	n.s.

Mean +/- S.D., a Control differs from MDD. Not statistically significant (p values greater than or equal to 0.05).

= Total sample size unless otherwise specified.

**Table 2:** Medications used by participants

	**Controls n = 36**	**MDD n = 70**	**P**
Medications	1.4 +/- 1.5	3.1 +/- 2.2	<0.0001
Antidepressants	0	1.1 +/- 0.7	n/a
Other Medications	0.8 +/- 0.8	1.0 +/- 1.2	n.s.
Other Psychotropic Medications	0 +/- 0.2 ^a^	0.3 +/- 0.7	0.0125
Vitamins/Minerals	0.2 +/- 0.4	0.3 +/- 0.6	n.s.
Analgesics	0.1 +/- 0.4	0.2 +/- 0.6	n.s.
Complementary/Alternative Medications	0.3 +/- 0.9	0.1 +/- 0.4	n.s.
Oral Contraceptives Users	38.9%	26.1%	n.s.

= Total sample size unless otherwise specified.

### Pain and associated symptoms

Pain location was distinct; the head and neck were the most common sites of pain. Pain at these locations was much more common in depressed (Table 3) and, within subjects with depression, in depressed subjects with atypical or melancholic episode subtypes (data not shown). Consistently, the proportion of pain-free subjects was significantly smaller in the depressed group. The intensity of pain was mild. Women with depression reported average values of approximately 2 (range 0–10) in all seven (general activity, mood, walking ability, normal work, relations with others, sleep, and enjoyment of life) interferences scales (data not shown). Therefore, in women with depression, pain interfered with the activities inquired, but only to a mild extent.

**Table 3:** Pain location and intensity

**Location of pain**	**Controls n = 36**	**MDD n = 70**	**P**
head/neck	16.7%	48.6%	**0.0008**
no pain	63.9%	42.9%	n.s.
legs	8.3%	22.9%	n.s.
back	11.1%	20%	n.s.
arms	5.6%	17.1%	n.s.
generalized	0%	4.2%	n.s.
**Brief Pain Inventory**	n = 19	n = 53	
Intensity of current pain	0.2 +/- 0.5	1 +/- 1.8	n.s.

Fatigue, anxiety, decreased concentration, and memory problems were more prevalent in subjects with depression (Table 4). A greater proportion of subjects with depression than controls (57% vs. 25%; P = 0.01) experienced four or more symptoms commonly associated with pain. Although the number of symptoms was much higher in subjects with MDD, controls were not totally symptom-free; more than one out of eight experienced insomnia, fear, weight changes, and fatigue. The vast majority of depressed subjects reported having at least one symptom, while nearly half of controls were symptom-free.

**Table 4:** Symptoms associated with pain

	**Controls n = 36**	**MDD n = 70**	**P**
Depression	8.3%	88.6%	**<0.0001**
Fatigue	13.9%	55.7%	**<0.0001**
Insomnia	25%	42.9%	n.s.
Anxiety	11.1%	40.0%	**0.0019**
Concentration/Memory problems	2.8%	31.4%	**0.0004**
Agitation	0%	27.1%	**0.0004**
Weight Changes	16.7%	21.4%	n.s.
Fear	16.7%	20.0%	n.s.
Nightsweats	2.8%	17.1%	n.s.
Change in appetite	5.6%	14.3%	n.s.
Constipation	5.6%	11.4%	n.s.
Nightmares	2.8%	11.4%	n.s.
Urinary Problems	2.8%	11.4%	n.s.
Drowsiness	2.8%	10.0%	n.s.
Shortness of Breath	2.8%	10.0%	n.s.
Disturbances in Intimacy/Sexual problems	5.6%	8.6%	n.s.
Diarrhea	2.8%	7.1%	n.s.
Rash, Pruritis	11.1%	7.1%	n.s.
Mouth Dryness, Lesions	2.8%	7.1%	n.s.
Numbness/Tingling	5.6%	7.1%	n.s.
Heart Irregularities	5.6%	5.7%	n.s.
Hiccoughs/Cough	2.8%	5.7%	n.s.
Nausea	5.6%	5.7%	n.s.
Visual Disturbances, Hallucinations	0%	5.7%	n.s.
Contractures, Spasms	2.8%	4.3%	n.s.
Dizziness	5.6%	4.3%	n.s.
Difficulty swallowing	0%	1.4%	n.s.
Edema, Swelling	5.6%	1.4%	n.s.
Mobility impairment	5.6%	1.4%	n.s.
Vomiting	5.6%	1.4%	n.s.
**Total Reporting Symptoms**	**52.8%**	**94.3%**	**<0.0001**
**Total Reporting No Symptoms**	**47.2%**	**5.7%**	**<0.0001**

### Relationship between pain intensity and clinical features of depression

Pain intensity, as measured by the Brief Pain Inventory, was weakly but significantly related with the current severity of depression, as measured by the HAMD (r^2 ^= 0.076; P = 0.04), and tended to be related with the current severity of anxiety, as measured by the HAMA (r^2 ^= 0.065; P = 0.07), and the number of episodes of depression (r^2 ^= 0.072; P = 0.09). No relationship was however observed between pain intensity and the total number of months of depression or the age at onset of depression.

### Measurements of stressors and quality of life: daily hassles and uplift scale, SF-36

**Figure 1:**
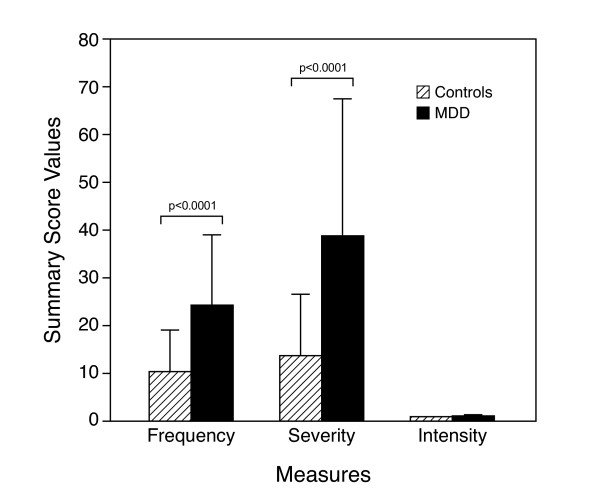
Daily Hassles Scale.

There were both quantitative and qualitative differences between groups in the Hassles Scale (Figure 1) and only qualitative differences in the Uplift Scale. Daily hassles occurred more frequently and more severely in women with depression. With the exception of concerns about body weight, there was also a qualitative difference in the type of hassles experienced by these two groups. In MDD subjects, the most common hassles were worries about physical appearance, misplacing things, and not having enough energy; the most common hassles in controls were preoccupations about health of a family member, not having enough time, and friends or relatives being far away. In contrast, both groups experienced daily uplifts to a similar extent (data not shown). Specifically, both women with depression and controls gained pleasure by reading and completing a task. In addition, uplifts specific to MDD subjects were laughing and visiting, phoning, or writing someone. The most common uplifts for controls included hugging and/or kissing and getting enough sleep.

**Figure 2:**
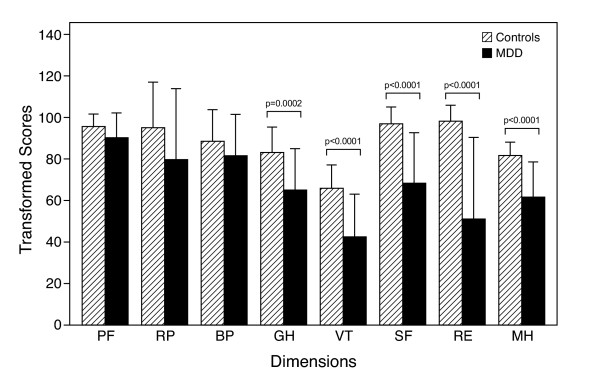
**SF-36 Questionnaire**. Physical Well-Being Domain: PF = Physical Functioning, RP = Role Physical, BP = Bodily Pain, GH = General Health; Emotional and Social Well-Being Domain: VT = Vitality, SF = Social Functioning, RE = Role Emotional, MH = Mental Health.

**Figure 3:**
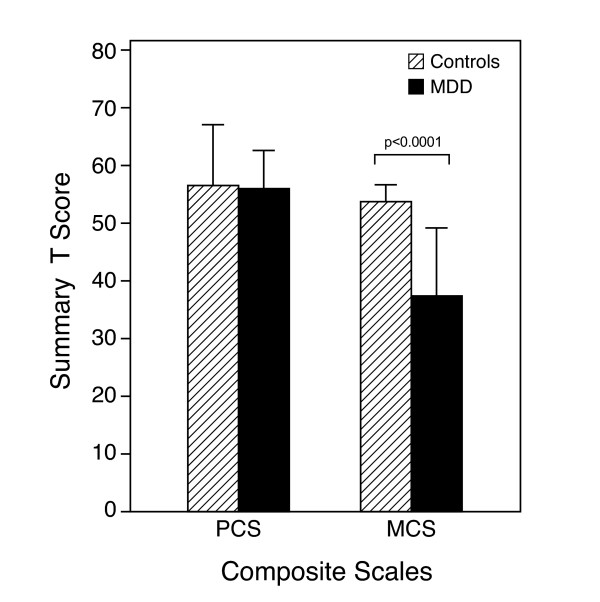
SF-36 Physical Composite Scale (PCS) and Mental Composite Scale (MCS).

SF-36 individual dimension scores and composite domains are depicted in Figure 2 and 3, respectively. In the emotional and social well-being domain, depressed subjects scored significantly lower than controls in all dimensions. In addition, in the physical well-being domain, the score of general health when compared to controls was abnormally low in depressed subjects. Figure 3 lower panel reports the aggregate domains for the physical composite summary and mental composite summary. Subjects with MDD were significantly affected in terms of the overall emotional and social well-being domain.

Depressed subjects reported that the most common aggravating factors of pain were mostly psychological or situational (e.g., anxiety, stress, fatigue, lack of sleep, menstrual cycle, overweight) in 21.4% of cases, whereas controls reported these factors as aggravating their pain only rarely (5.6%, p = 0.03). Rather, controls frequently tended to describe physical factors (e.g., increased activity, bending, poor posture, standing, walking, daily activities, lifting, uncomfortable shoes, prolonged squatting) as causing their pain to worsen (27.8%). Previous treatments and factors found to alleviate pain in both groups were found to be similar. These included practitioner-administered treatments (e.g., medications, surgery, massage therapy, chiropractic, physical therapy, acupuncture, psychotherapy) and self-administered treatments (e.g., heat, ice, stretching, exercise, decrease in activity, rest, deep breathing). The most common words used by depressed and control subjects to express their pain were in terms of somatic pain terminology, such as musculoskeletal pain descriptors (data not shown). The prevalence of low bone mass was significantly greater in women with MDD (data not shown). All subjects were unaware of their low bone mass, and none of these subjects had fractures.

### Cytokine and hormonal measurements

**Figure 4:**
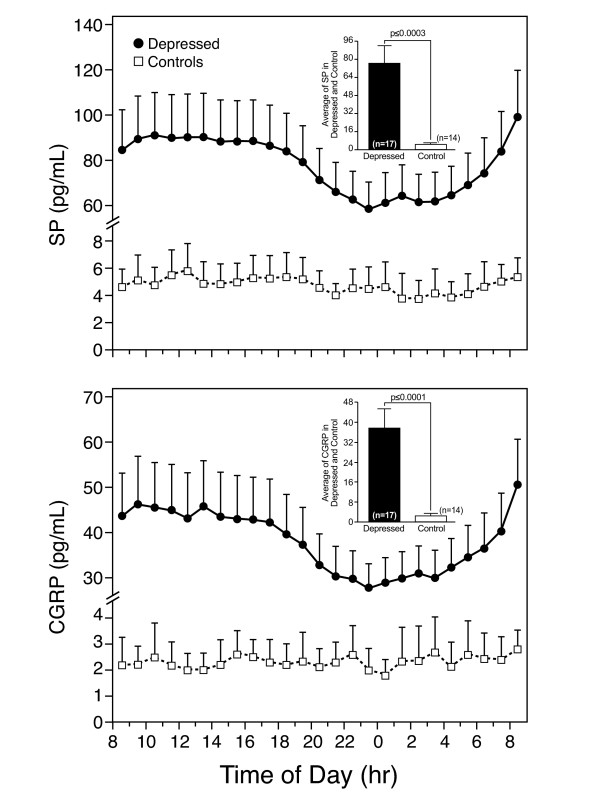
**Plasma levels of Substance P (SP) (upper panel) and calcitonin-gene-related-peptide (CGRP) (lower panel)**. As indicated in the insets, mean 24-h levels of SP and CGRP were lower in women with depression compared to controls.

Women with depression (n = 17) had higher mean circulating levels of SP and CGRP than controls (N = 14) (Figure 4). Both SP and CGRP exhibited a 24-h single cosinor rhythm in women with depression which was remarkably similar to controls; the zenith occurred at 12:24 and 12:15 respectively, and the nadir at 00:24 and 00:15, respectively. SP (zenith: 13:50, nadir: 01:50) exhibited a significant rhythm in controls whereas no significant rhythm in CGRP was observed in controls.

## Discussion

Premenopausal women with depression had a higher prevalence of pain than generally reported in the literature. In addition, these subjects experienced a lower quality of life, especially in the psychological domain, and complained more of negative daily stressors compared to controls. SP and CGRP, two pain-related neuropeptides, were higher around the clock in depressed subjects compared to controls.

Half of the women with MDD in this sample reported mild pain and headaches associated with fatigue, anxiety, and concentration and memory problems. The observed prevalence of pain is remarkable, given the relatively young age of this sample; usually pain is an indicator of depression in the elderly [21]. Almost half of the subjects with MDD complained also of insomnia. Sleep disturbances are known to increase pain sensation. We observed an association between pain intensity and severity of depression and anxiety, which is intriguing and points towards a possible link between these two conditions. Consistent with the complex and multifactorial nature of pain, severity of depression accounted only in part for pain intensity. To further complicate matters, aggravating factors of pain such as fatigue and insomnia are also part of the syndromic definition of major depression.

Women with depression reported a lower quality of life. They scored lower than controls in the emotional and social well-being domain of the SF-36, as well as in one dimension of the physical well-being domain, general health. Thus, they described themselves as being less vital, affected emotionally and impaired in their social life. The impact of their depression on quality of life was comparable to that reported in subjects with breast cancer or morbid obesity [16,22].

The role of major stressors in the clinical course of MDD has been widely evaluated, but the effects of small, positive or negative, daily life events have not been fully explored. Women with depression had a heightened perception of negative daily stressors. They were preoccupied with self-dependent worries such as physical appearance, misplacing or losing things, and not having enough energy. In addition, their hassles clearly differed in nature from those of controls, who had worries that were person-dependent and mostly focused on health of a family member, not having enough time, and friends or relatives being far away. Therefore, subjects with depression, as previously reported in subjects with chronic fatigue syndrome and fibromyalgia, seem not to adapt well to their daily worries and concerns, have problems with their self-image, and struggle with various daily hassles [23]. Chronic, severe stress and subsequent activation of the hypothalamic-pituitary-adrenal axis are associated with augmented pain sensitivity [24]. The potential role of less severe daily stressors on the development and maintenance of pain syndromes remains to be determined.

Among the various hormonal and immune alterations described in subjects with MDD [25], SP and CGRP may amplify pain symptoms. There are very few studies of SP or CGRP in subjects with depression [26,27]. SP is a neurokinin expressed in the peripheral and central nervous systems in serotoninergic and noradrenergic pathways involved in both depression and pain. CGRP potentiates the release of SP and promotes the transmission of pain stimuli. CGRP levels were increased in subjects with depression, both in plasma and in the CSF [27]. Consistent with a previous report [26], 24-hour circulating levels of SP and CGRP, were higher in women with depression compared to controls. Interestingly, the secretion of SP and CGRP over 24 hours showed a similar diurnal variation, with higher circulating levels during the daytime and lower levels at night. Consistent with their mild depression, the women with MDD were eucortisolemic [28]. Our findings suggest that SP and CGRP may be useful biological markers in women with MDD.

We recently reported that these women with MDD also exhibited low bone mass [29]. In addition, they had increased abdominal fat and higher levels of plasminogen activator-1 and Factor VIII [13]. The collective evidence generated by the P.O.W.E.R. study suggests a phenotype of MDD characterized by somatic symptoms and pain and further associated with medical consequences such as increased fractures and greater cardiovascular risk.

Our study included a large and homogeneous sample. The study was however limited by its cross-sectional design and by the subjective nature of pain. The physiological meaning of elevation in SP and CGRP is unclear. As the majority of these patients were taking antidepressants, it was not possible to assess the potential role of antidepressants in modulating pain intensity.

## Conclusion

In summary, we found a high prevalence of pain of mild intensity, increased neuropeptide levels, increased everyday stress, and diminished quality of life in young women with depression. Women with depression should be clinically screened for pain and patients complaining of pain, especially if not clearly attributable to an identifiable organic process, should be evaluated for depression. Future research should establish the role of MDD in the natural history of pain, attempt to characterize the biological mechanisms predisposing to pain, and determine whether men with depression also have increased prevalence of pain symptoms and increased SP and CGRP circulating levels.
